# Effectiveness of a Web-Based Communication Education Program in Improving Knowledge and Self-Efficacy in Family Caregivers of Nursing Home Residents: A Quasi-Experimental Study

**DOI:** 10.1097/jnr.0000000000000748

**Published:** 2026-05-11

**Authors:** Ching-Hui HSIAO, Yu-Ting CHEN, Wann-Yun SHIEH, Hsiu-Hsin TSAI

**Affiliations:** 1 Department of Nursing, Meiho University, Pingtung, Taiwan; 2 School of Nursing, College of Medicine, Chang Gung University, Taoyuan, Taiwan; 3 Department of Computer Science and Information Engineering, Chang Gung University, Taoyuan, Taiwan; 4 Department of Psychiatry, New Taipei Municipal Tucheng Hospital, New Taipei City, Taiwan

**Keywords:** communication skills, web-based education, self-efficacy, nursing home, older adults

## Abstract

**Background:**

Effective communication between family members and nursing home residents is vital. However, limited research has been conducted, especially in Asia, regarding the effectiveness of web-based communication programs in improving the communication efficacy of family members of nursing home residents.

**Purpose:**

In this quasi-experimental study, the effectiveness of a 4-week, nurse-led web-based communication education program (WBCEP) was evaluated in terms of improving the communication skills-related knowledge and self-efficacy of the family members of nursing home residents.

**Methods:**

A total of 68 family members of older adult residents of medium-sized facilities (30 < *n* < 90) were enrolled as participants. The experimental group (*n* = 34) participated i*n* the WBCEP, while the control group (*n* = 34) did not. Data were collected at baseline and at 1 and 2 months after baseline. Generalized estimating equations were used to analyze the longitudinal effectiveness of the program.

**Results:**

The experimental group improved significantly more in both communication skills knowledge and self-efficacy scores, including lagged effects, than the control group. Moreover, the experimental group reported high levels of learning satisfaction.

**Conclusions/Implications for Practice:**

The innovative nurse-led WBCEP implemented in this study was shown to be effective in enhancing communication skills knowledge and self-efficacy in family members of nursing home residents, contributing to overall efforts to improve family-centered care.

## Introduction

The convergence of demographic and societal changes, including increased life expectancy, lower birth rates, smaller family structures, and industrialization, has led to a rising number of older adults in Taiwan requiring long-term care in nursing homes (NHs; Ministry of Health and Welfare, 2022). Transitioning to institutional care presents emotional and relational challenges for both residents and their family members. Thus, maintaining strong family involvement, especially through effective communication, is essential to preserving residents’ social support and promoting their well-being (Donovan & Blazer, 2020; Gautam et al., 2024).

Effective communications between family members and NH residents strengthen emotional bonds, reduce misunderstandings, and support smoother adjustment to the NH environment (Ghazavi et al., 2016; Tasseron-Dries et al., 2021). However, numerous barriers hinder these communications. Many NH residents experience age-related hearing or visual impairments, and a large proportion are diagnosed with dementia, all of which complicate verbal and nonverbal interactions (Boamah et al., 2021; Dawes et al., 2021; Kao et al., 2022). These challenges may negatively impact both caregiver confidence and the quality of family visits, especially in situations marked by reduced social contact.

In Taiwan, cultural values such as filial piety shape caregiving expectations and encourage frequent family visits (Tsai & Tsai, 2012). Yet, despite these cultural norms, communications remain a challenge, with nearly 44% of family caregivers reporting difficulties in interacting effectively with residents (Wu & Rong, 2020). These challenges are particularly pronounced when residents are experiencing cognitive decline. Chen et al. (2025) further emphasized that caregivers of patients with dementia in institutional settings often experience significant emotional and physical burdens, underscoring the need for accessible, structured support systems tailored to the specific needs of family caregivers.

Communication skills training has been shown to improve interactions between NH staff and residents, particularly those with cognitive or sensory impairments (Sprangers et al., 2015). While this training has proven effective for professional caregivers, its potential application to family members remains underexplored (Lindig et al., 2024). A recent review found that most communication programs focus on professional caregivers in Western settings and vary widely in content and format (Machiels et al., 2017), highlighting a gap in programs tailored for family caregivers, especially within East Asian cultural settings.

Web-based educational programs present a promising and scalable solution for bridging this gap, with related platforms offering flexible and accessible training options that accommodate the needs of various schedules and geographic locations (Cristancho-Lacroix et al., 2015). Ji and Chi (2024) found higher levels of eHealth literacy to be associated with greater health knowledge and self-efficacy, which suggests digitally literate caregivers may be well-positioned to benefit from web-based training, supporting the integration of digital platforms into caregiver education. In addition, Shao et al. (2024) demonstrated that digital interventions incorporating interactive, motivational, and user-centered features significantly enhance self-management efficacy in chronic illness care.

The above findings highlight the importance of thoughtful design in the development of digital education tools. Therefore, the aim of this study was to evaluate the effectiveness of a 4-week, nurse-led Web-Based Communication Education Program (WBCEP) in improving communication knowledge and self-efficacy among a sample of family members of NH residents. The study hypotheses were that using the WBCEP would result in: (1) significantly better communication-related knowledge in the experimental than the control group, and (2) significantly better communication self-efficacy with residents in the experimental than the control group.

## Methods

### Research Design

The institutional coordination required to implement the intervention limited the practicality of randomization. Thus, a nonrandomized controlled trial design was adopted to support voluntary participation and ensure adherence to ethical standards.

### Setting and Sample

Due to cost considerations, two large-scale NHs were selected based on size and accessibility to the researchers. To avoid contamination, the experimental and control groups were recruited, respectively, from one of the NHs only. One unit in each NH was assigned for recruitment, and the family members of residents were invited to participate. Family caregivers who were an adult (≥ 20 years old) family member of a resident in the selected NH were recruited as participants. The inclusion criteria were: (a) family member of an NH resident, (b) ability to communicate in Mandarin or Taiwanese, (c) 20 years old or older, and (d) consent to participate. Otherwise qualified family members with visual, auditory, or cognitive impairments were excluded. This study was conducted from September 2023 to February 2024.

### Web-Based Communication Education Program

The WBCEP used in this study is a 4-week web-based program designed to help family members understand the importance of communicating with older adults in NHs (Kang & Seomun, 2018). This program is also designed to help family members recognize common communication barriers and learn how to communicate effectively with older adult NH residents (including managing sensory impairments, cognitive decline, and techniques to address forgetfulness and feelings of being dismissed). The web-based format allows users to access the program at their convenience on various devices such as mobile phones, tablets, and computers (Table 1).

**Table 1:** Main Content Items and Communication Subdomains in the Web-Based Communication Education Program

Communication Skill Stage	Subdomain	Education Content
Introduction	Understanding communication	• Factors related to successful communication • The importance of listening and nonverbal communication (gestures, facial expressions, and body movements) • Factors affecting communication between families and older adults nursing home residents
Communication difficulties	Communication difficulties in older adults	• Introduction • Common communication challenges vision impairment hearing impairment cognitive impairment mental health issues • Case video viewing
Communication needs	Older adults/families	• Recognizing the psychological and emotional needs of older adults • Understanding the communication needs of the older adults • The importance of family involvement of communication
Communication strategies	Effective communication strategies	• Introduction How to communication with older adults • Communication strategies • Enhancing active listening • Verbal communication skills • Improving nonverbal communication • Adjusting communication for sensory impairment • Managing communication for cognitive impairment • Emotional and psychological support • Case video viewing • Discussion
Evaluation	Evaluation Feedback	• Course survey • Posttest • Self-assessment • Q&A session

The didactic methods used in the program include lectures, group discussions, and role-play. A tailored course website was developed using WordPress, which is a versatile content management system that enhances functionality through various plugins and themes. Many themes were designed for educational purposes, offering a professional and user-friendly experience. The program is designed to be highly accessible and easily navigated, even by users with limited technical expertise (Lin et al., 2017; Novokmet et al., 2023).

Communication scenarios in NHs were recorded as role-playing videos to demonstrate common communication problems and create a sense of immediacy via situational simulations. The course content, prepared and presented in Microsoft PowerPoint format, incorporates voice recordings and is published as multimedia courseware on YouTube. YouTube is a primary medium for knowledge dissemination, allowing users to search for and watch health-related videos to enhance understanding (Huang, 2021; Shoufan & Mohamed, 2022). Through YouTube, users may upload, search, and watch videos using relevant keywords of interest. Each video provides information such as the uploader, video details, view count, upload date, and comments (Chung, 2023; Huang, 2021).

### Instruments

Instruments were used in this study to gather data on sociodemographic characteristics, communication skills knowledge, and communication self-efficacy. The collected sociodemographic characteristics included the participant’s gender, whether family members and residents used communication software to stay in contact, prior experience with online learning, previous participation in related communication education courses, and whether communication challenges arose due to resident sensory (hearing or vision) or cognitive impairments.

The communication skills knowledge scale was developed by the authors based on the relevant literature (Chen, 2021; Lee, 2009; Wang et al., 2011). This scale is designed to measure the relevant communication skills knowledge that family members of NH residents possess in the context of interacting with older adults. The scale comprised 21 true/false items addressing the realms of communication barriers in older adults, verbal and nonverbal communication, and communication techniques.

Self-efficacy was assessed in this study using a self-developed scale adapted from the Self-Efficacy in Communication Scale (Axboe et al., 2016) and Communication Self-Efficacy Scale (Tatsumi et al., 2012). These two scales were revised and modified based on Bandura’s self-efficacy theory to include items specifically relevant to family members communicating with NH residents. The final scale included 16 items scored from 1 (*no confidence*) to 10 (*high confidence*), with higher total scores reflecting greater self-efficacy in communicating with older adult residents. The scale had a Cronbach’s alpha of .98 in this study.

A post-learning satisfaction questionnaire was constructed based on a framework for teaching, learning curriculum, environment, and outcomes developed by Wang (2003); the design and presentation of teaching materials, system functions, and overall learning perceptions developed by Huang (2004); and the learning satisfaction dimensions of course content, delivery, teaching materials, atmosphere, and course quality developed by Urdan (1997). The questionnaire included 20 items in four parts: (1) communication skills course (seven questions), (2) web-based course materials (six questions), (3) web-based communication health education course after the course (four questions), and (4) overall feelings of the web-based communication health education course process (three questions). Responses were measured on a 5-point Likert scale ranging from 1 (*very dissatisfied*) to 5 (*very satisfied*). This questionnaire served as the post-learning satisfaction survey for the experimental group.

All of the tools and scales were evaluated by experts for content validity. The values for the item content validity index and scale content validity index/universal agreement were both 1.0. Communication skills knowledge was further analyzed using Kuder–Richardson reliability to assess difficulty and discrimination, yielding a Kuder–Richardson formula 20 value of .76, indicating acceptable reliability.

### Procedures

After securing all of the approvals required to conduct this study, the research procedures were posted at the entrance of both participating nursing homes, and potential participants were contacted directly by the research team. The participants in the experimental group were allowed access via smartphone, tablet, and computer platforms to the 4-week WBCEP. Research staff monitored participant progress on a weekly basis and encouraged continued use of the program, including engagement with the platform’s discussion board. They also reviewed usage log records to verify participants were actively using the program and receiving sufficient exposure to its content. Conversely, the participants in the control group received routine care without additional educational support. Study data were collected at three time points, with baseline data obtained soon after caregivers agreed to participate and subsequent data collected at, respectively, 1 and 2 months after the baseline measurement. Data from both groups were collected with assistance from the research staff via paper surveys, QR code scans, or email. Due to the nature of the intervention, blinding participants or study personnel was not feasible. However, the outcome analysis was performed by an independent statistician who was blinded to group assignments.

### Ethics

This study was approved by the institutional review board (no. 202100609B0A3). Clinical trial registration number: NCT06448221. Furthermore, the authors received permission to conduct this study from the directors of both nursing homes before data collection. Before collecting data, the first author explained the study purpose and procedures to the participants, informed them they could withdraw at any time and/or refuse to answer any questions, and guaranteed the confidentiality of their data.

### Data Analysis

All of the data were coded and analyzed using SPSS for Windows version 28.0 (IBM Corp., Armonk, NY, USA). Before data analysis, all of the variables were reviewed for accuracy and missing values. Differences in caregiver and patient characteristics between the experimental and control groups were assessed using independent sample *t-*tests and chi-square tests. Generalized estimating equations were employed to compare the degrees of change in each variable between the control and experimental groups over the three time points. Statistical significance was set at *p* < .05.

## Results

Sixty-eight family members of older adult long-term care institution residents were enrolled as participants. The average ages in the experimental (*n* = 34) and control groups (*n* = 34) were 53.56 ± 8.90 and 48.88 ± 10.59 years, respectively. Most of the participants were female (69.1%) and were primarily the children of the residents (64.7%). Most had a high school (vocational) or college-level education (88.2%), were employed (67.6%), and had lived with others before the residents were placed in care (57.4%). Moreover, most had visited the residents in person at the institution (64.7%), did not use communication software (e.g., LINE) to contact the residents (61.8%), had web-based class experience (63.2%), and had not attended communication education courses (76.5%). Also, most reported difficulties in communicating with the residents (73.5%), the majority of whom were affected by cognitive impairments. No significant between-group differences were detected in terms of demographic characteristics, indicating a high degree of intergroup homogeneity (Table 2).

**Table 2:** Demographic Characteristics of the Participants (*N* = 68)

	Experimental Group (*n* = 34)	Control Group (*n* = 34)		
Variable	*n* (*%*)	*n* (*%*)	*t/*χ^2^	*p*
Age (mean and *SD*)	53.56 (8.90)	48.88 (10.59)	2.15	.530
Sex			1.72 ^a^	.189
Male	13 (38.2)	8 (23.5)		
Female	21 (61.8)	26 (76.5)		
Relationship with residents			1.94 ^a^	.925
Spouse/daughter-in-law	5 (14.7)	5 (14.7)		
Child	21 (61.8)	23 (67.6)		
Grandchildren	5 (14.7)	5 (14.7)		
Nephew/niece	1 (2.9)	0 (0.0)		
Other (son-in-law, friend)	2 (5.9)	1 (2.9)		
Educational background			6.10 ^a^	.192
Elementary/junior high school	3 (8.8)	5 (14.7)		
High school (vocational)/college or above	31 (91.2)	29 (85.3)		
Currently employed			3.44 ^a^	.752
No	12 (35.3)	10 (29.4)		
Yes	22 (64.7)	24 (70.6)		
Lived with resident before relocation			0.06 ^a^	.806
No	15 (44.1)	14 (41.2)		
Yes	19 (55.9)	20 (58.8)		
In-person visit frequency			7.18 ^a^	.028
Often	27 (79.4)	17 (50.0)		
Sometimes	7 (20.6)	15 (44.1)		
Seldom	0 (0.0)	2 (5.9)		
Use communication software with the resident			3.38 ^a^	.496
No	19 (55.9)	23 (67.6)		
Yes	15 (44.1)	11 (32.4)		
Web-based classes experience			0.57 ^a^	.451
No	11 (32.4)	14 (41.2)		
Yes	23 (67.6)	20 (58.8)		
Communication education courses experience			1.31 ^a^	.253
No	28 (82.4)	24 (70.6)		
Yes	6 (17.6)	10 (29.4)		
Communication problems with elderly residents			2.72 ^a^	.099
No	12 (35.3)	6 (17.6)		
Yes	22 (64.7)	28 (82.4)		
Communication barriers with elderly residents (multiple choice)				
Hearing impairment	7 (33.3)	11 (45.8)		
Visual impairment	2 (9.5)	0 (0.0)		
Speech impairment	7 (33.3)	3 (12.5)		
Cognitive impairment	12 (57.1)	14 (58.3)		

^a^ Chi-square.

The average baseline score for communication skills knowledge in the experimental group was lower than in the control group. After the intervention, the experimental group scored higher for communication skills knowledge than the control group on both the 1-month and 2-month posttests (Table 3). Also, the average baseline score for self-efficacy in the experimental group was lower than in the control group. Similarly, after the intervention, the experimental group scored higher for self-efficacy than the control group on both the 1-month and 2-month posttests (Table 3).

**Table 3:** Communication Skills Knowledge and Self-Efficacy Scores at Baseline and 1- and 2-Month Posttest

	Total	Experimental Group	Control Group
Statistical Value	Mean (*SD)*	Mean (*SD)*	Mean (*SD)*
Communication skills knowledge			
Baseline	16.49 (2.34)	16.36 (2.52)	16.63 (2.15)
1-month	17.87 (2.20)	18.91 (1.64)	16.73 (2.19)
2-month	18.05 (2.11)	19.21 (1.26)	16.77 (2.21)
Self-efficacy			
Baseline	101.57 (30.34)	101.27 (28.86)	101.90 (32.39)
1-month	106.97 (27.52)	111.85 (22.96)	101.60 (31.32)
2-month	107.03 (27.63)	111.88 (22.99)	101.70 (31.50)

### Effectiveness of the WBCEP on Families of NH Residents

The effects of the WBCEP on communication skills knowledge scores are summarized in Table 4. Knowledge of communication skills improved significantly in the experimental group at both the 1st and 2nd months posttest (β = 2.44, *p* < .001 and β = 2.71, *p* < .001, respectively), while no significant change over time was noted in the control group.

**Table 4:** Generalized Estimating Equations Analysis of Group and Time on Communication Skills Knowledge and Self-Efficacy

Variable	β	*p*
**Communication skills knowledge**		
Group (experimental: control)	−0.27	.642
Time (reference group: pretest)		
1-month posttest	0.10	.463
2-month posttest	0.13	.365
Group × time (reference: control × time)		
Experimental × 1-month posttest	2.44	< .001***
Experimental × 2-month posttest	2.71	< .001***
**Self-efficacy**		
Group (experimental: control)	−0.62	.935
Time (reference group: pretest)		
1-month posttest	0.30	.469
2-month posttest	−0.20	.565
Group × time (reference: control × time)		
Experimental × 1-month posttest	10.87	.002**
Experimental × 2-month posttest	10.80	.002**

**p < .05. ***p < .001.

The effects of the WBCEP on self-efficacy scores are summarized in Table 4. Self-efficacy regarding communication skills improved significantly in the experimental group at both the 1st and 2nd month posttests (β =10.87, *p* = .002 and β =10.80, *p* = .002, respectively), while no significant change over time was noted in the control group.

### Satisfaction With the WBCEP

To assess satisfaction with the WBCEP, a 5-point Likert satisfaction questionnaire was conducted on the experimental group after the course. The highest average score was for overall program experience (4.67 ± 0.47), while the lowest average scores were for “The communication skills knowledge course has enhanced my ability to identify communication barriers with elders” (4.48 ± 0.50), “The web-based learning materials have improved my learning effectiveness” (4.48 ± 0.56), and “The web-based learning materials meet my needs,” (4.48 ± 0.56). Notably, all questionnaire items earned average scores above 4.5 (90%), demonstrating strong satisfaction with all aspects of the WBCEP.

Further analysis of satisfaction scores revealed that overall program experience had the highest average (4.61 ± 0.04), followed by post-course perceptions (4.59 ± 0.03) and knowledge of communication skills (4.57 ± 0.05) items. The lowest average score was earned by the online learning materials item (4.53 ± 0.04). As above, all questionnaire items earned average scores above 4.5 (90%), again demonstrating strong satisfaction with all aspects of the WBCEP.

## Discussion

After the 4-week WBCEP intervention, the level of communication skills knowledge was significantly higher in the experimental group than in the control group. This result aligns with the systematic review and meta-analysis on web-based nursing education conducted by Kang and Seomun (2018), who demonstrated the effectiveness of short-period (2–4 week) interventions. However, Pinquart and Sörensen (2006) emphasized that for an intervention to be truly effective, participants must practice the skills during training and apply the learned skills and knowledge to verify their effective transmission. To increase participation in this study, burdens were reduced by considering the time, distance, and lifestyle constraints of family members, resulting in no time available for participants to practice learned skills.

This study contributes to a growing body of research that supports the effectiveness of short-term, low-burden, web-based interventions in enhancing communication-related knowledge and self-efficacy among family caregivers in long-term care settings. The results underscore the potential of digital education to support caregiver roles, particularly when in-person training is limited. While the intervention was successful in improving knowledge, the practical application of skills in real-life caregiving scenarios was not fully addressed. To bridge this gap, future programs may incorporate AI-driven interactive components such as personalized learning paths, real-time feedback, and simulation-based training. These technologies offer scalable and adaptive solutions for further enhancing caregiver engagement and skill development and, ultimately, improving communication outcomes between families and nursing home staff.

Although the control group did not participate in the WBCEP, the average communication skills knowledge score of the control group participants increased between baseline and both posttests. However, these increases were not significant and may be attributable to external factors, for example, self-learning using related online resources or exposure to relevant information after the baseline assessment.

Furthermore, self-efficacy increased significantly at both the 1-month posttest (compared with baseline) and the 2-month posttest (compared with the 1-month posttest), with these increases greater than those observed in the control group. While the communication skills course increased self-efficacy scores compared with those at baseline, the initial baseline scores were relatively high. This aligns with Axboe et al. (2016), which also evaluated changes in health care provider self-efficacy after communication skills training, finding that, when test scores are already high, participants tend to perform well regardless of skill level, potentially leading to a ceiling effect. However, in this study, the high baseline scores did not completely limit improvement, as the course appeared to address specific practical challenges that the participants were not previously equipped to handle. This suggests that the intervention effectively enhanced self-efficacy in areas relevant to real-world communication barriers, even among initially confident participants. The initial confidence level may have underestimated the actual difficulties posed by communication barriers when interacting with older adults facing communication challenges.

These findings also resonate with Shao et al. (2024), which demonstrated that well-designed, user-centered digital interventions can enhance self-management efficacy in patients with chronic illness. While their study focused on patient populations, the principles underpinning their intervention, such as interactivity, personalization, and accessibility, may be analogously applied to caregiver education. The findings of this study support this broader application by showing that digital platforms, when appropriately structured, can effectively increase self-efficacy in caregivers to address practical and emotional challenges.

Participants in the experimental group generally reported that the learning experience met their expectations, as reflected in the high overall satisfaction scores, suggesting the web-based instructional materials and course content developed for this study met the expectations of the families of NH residents. In addition, the use of PowerPoint presentations with voice-overs that were recorded as videos and segmented into three YouTube videos linked to WordPress allows learners to proceed at their own pace through segmented learning. The features of the website, including detailed learning objectives, diverse images, animations, relevant video links, and a discussion area, received positive feedback by offering asynchronous learning options that took into consideration the unique circumstances of family members. Notably, to enhance accessibility, participants were not required to log into the website, reducing barriers to online navigation. However, this decision also limited the ability to track participants’ usage data, potentially introducing bias in evaluating the effectiveness of content usage. Future research should incorporate methods to track engagement and analyze its relationship with outcomes.

Although the results of this study indicate that the WBCEP effectively enhances both communication knowledge and self-efficacy in family caregivers, residents’ psychosocial outcomes, such as anxiety, stress, and social isolation, were not considered. Although beyond the primary scope of the intervention, these outcome variables are closely linked to psychological well-being in residents of long-term care facilities. In cultures that emphasize filial piety, improving the communication skills of family caregivers may foster more effective interactions with residents, potentially reducing the psychological burden associated with communication barriers. Therefore, future research should incorporate relevant mental health indicators to assess the potential impact of improved caregiver communication on the psychological outcomes of residents.

Lastly, the inclusion criteria did not consider the cognitive or sensory status of nursing home residents, reflecting the current reality of caregiving in institutional settings. While variations in condition among residents may introduce potentially confounding effects, the observed improvements in caregiver communication self-efficacy suggest the intervention has broad applicability across diverse caregiving scenarios. Nonetheless, this issue must be addressed with greater precision in future research, with participants stratified based on the cognitive and sensory functioning levels of the care recipient to more accurately assess intervention effectiveness and to inform the development of tailored communication strategies.

### Conclusions

The results of this research demonstrate that WBCEPs can improve the communication skills, knowledge and self-efficacy of program participants significantly. Implementing these programs in NHs can enable family members to learn flexibly through integrating courses into their schedules in a manner that minimizes interference with work and personal responsibilities.

### Author Contributions

Study conception and design: All authors

Data collection: CHH, WYS, HHT

Data analysis and interpretation: CHH, YTC

Drafting of the article: All authors

Critical revision of the article: HHT
